# Antibiotic artificial bone implantation for the treatment of infection after internal fixation of tibial plateau fractures

**DOI:** 10.1186/s12891-022-06112-z

**Published:** 2022-12-31

**Authors:** Haotian Hua, Lei Zhang, Zairan Guo, Wenlong Zhong, Jiangfei Chen, Jiangang Guo, Yang Zhang, Peijian Tong, Xinwei Wang

**Affiliations:** 1 The First School of Clinical Medicine, Zhejiang Chinese Medical University, Hangzhou, China; 2 Research and Treatment Center of Bone and Joint Infections, Luoyang Orthopedic-Traumatological Hospital of Henan Province (Henan Provincial Orthopedic Hospital), Luoyang, China; 3 The First Affiliated Hospital of Zhejiang Chinese Medical University (Zhejiang Provincial Hospital of Traditional Chinese Medicine), Hangzhou, China

**Keywords:** Antibiotic artificial bone, Tibial plateau fractures, Internal fixation, Infection

## Abstract

**Objective:**

To explore the clinical effect of antibiotic artificial bone (Calcium phosphate) in the treatment of infection after internal fixation of tibial plateau fractures.

**Methods:**

We retrospectively reviewed the clinical data of 32 patients with infection after internal fixation of tibial plateau fractures treating from March 2010 to October 2021. There were 18 males and 14 females, aged from 23 to 70 (average 49.66 ± 10.49), 19 cases of the left side and 13 cases of the right side. Among them, 7 cases were open fractures with initial injury and 25 cases were closed fractures. On the basis of thorough debridement and implanting antibiotic artificial bone, the internal fixation of 18 patients were tried to be preserved and the internal fixation of 14 patients were removed completely. In order to provide effective fixation, 14 patients also received external fixation. Postoperative wound healing, infection control, Hospital for Special Surgery knee scores (HSS), related inflammatory indicators and bone healing time were recorded and followed up.

**Results:**

Thirty-two patients were followed up for 12 ~ 82 months (average 36.09 ± 19.47 months). The redness, swelling and pain of pin site occurred in 2 patients, which returned to normal after applying antibiotics and continuous dressing change. One patient retained the internal fixation during the first-stage operation. Redness and swelling of incision, subcutaneous undulation occurred after two months. In order to avoid the recurrence of infection, the internal fixation was removed completely and antibiotic artificial bone was filled again. The infection was controlled and fracture healed. Four patients’ wounds could not be closed directly due to soft tissue defect and was covered with skin flap. After the first-stage operation, 12 patients received second-stage autologous iliac bone grafting due to residual bone defects and poor healing of the fracture end. The bone healing time was 4 ~ 16 months (average 7.31 ± 2.79 months). Inflammatory indicators including CRP, ESR, and WBC returned to normal levels within 2 ~ 10 weeks (average 4.97 ± 2.58 weeks). The HSS of all patients were 54 ~ 86 points (average 73.06 ± 8.44 points) at the last follow-up.

**Conclusion:**

Implantation of antibiotic artificial bone, retention or removal of internal fixation according to infection and fracture healing, application of external fixation timely is an effective method to treat infection after internal fixation of tibial plateau fractures, which can control infection effectively and promote functional recovery.

## Introduction

Tibial plateau fractures are the most common intra-articular fractures, which often cause the damage of soft tissues in the joint. Moreover, the tibial plateau is the most important weight-bearing area of the knee joint. Improper treatment can cause severe damage to the knee joint function, which will affects the quality of patients’s life greatly [1, 2]. Open reduction and internal fixation(ORIF) can not only stabilize the fracture site and achieve anatomical reduction, but also reconstruct the collapsed articular surface and restore the normal force line of lower limbs. Therefore, it has become the golden standard for the treatment of tibial plateau fractures [3]. Because the soft tissue in front of the tibia is weak, open reduction will also aggravate the damage of soft tissues. Therefore, infection of tibial plateau fractures after ORIF often occurs, and it has been reported in the literature that the incidence reaches 2.6% ~ 45%, which are 4 ~ 5 times higher incidence of infection after fractures than other parts [4].

At present, there are no standard treatment strategies and methods for infection after internal fixation of tibial plateau fractures, and there are few relevant clinical reports [5, 6]. Current treatment options include radical debridement, irrigation, flap coverage, induced membrane technique. However, these methods have some disadvantages, including long treatment period, recurrennt infection, high demand for autologous bone [7–9]. Debridement and the application of antibiotics have always played an important role in the treatment of bone infections. The topical application of antibiotics has been valued increasingly. Calcium phosphate loaded with antibiotics leads to anti-infection and repairing bone defects. Previous clinical researches have also verified the effectiveness of this method in the treatment of osteomyelitis and bone defects [10]. We think that the purpose of treating infection after ORIF of tibial plateau fractures is to control infection, promote fracture healing, maintain knee joint function, and improve the quality of patients’s life. Based on this concept, we used antibiotic artificial bone and applied external fixation in time to treat infection after ORIF of tibial plateau fractures, while the removal of internal fixation is determined by fracture healing and degree of infection. The purpose of this study was to assess the effectiveness of this method.

## Methods

In this study, the clinical data of 32 patients diagnosed with infection after internal fixation of tibial plateau fractures and treated at our department from March 2010 to October 2021 were retrospectively analyzed. The study was approved by the Luoyang Orthopedic Hospital’s ethical review committee (KY2018-001–01). Informed consent was obtained from all the patients.

The inclusion criteria were as follows: ⑴Meet the diagnostic criteria for postoperative infection of tibial plateau fractures after internal fixation [11]. ⑵Patients received antibiotic artificial bone implantation. ⑶The patients were older than 18 years. The exclusion criteria were as follows: ⑴The patient had an underlying disease that affects the efficacy and prognosis significantly. ⑵The patients were allergic to vancomycin or gentamicin. ⑶The patient’s medical records were missing or follow-up was incomplete. The diagnosis of infection after internal fixation of tibial plateau fractures was based on at least one of the following conditions [11]: ⑴The fistula and sinus that connected with implant or bone tissue; ⑵Pus was found around the implants during the operation; ⑶The bacterial culture of the suspected infected tissue was positive; ⑷Histopathological examination confirmed the presence of pathogenic microorganisms.

### Surgical procedures

#### Preoperative treatment

After admission, all patients underwent relevant laboratory tests, including blood routine, erythrocyte sedimentation rate, C-reactive protein and bacterial culture. The results showed that: 8 cases of staphylococcus aureus, 4 cases of staphylococcus epidermidis, 2 cases of enterobacter cloacae, 1 case of klebsiella pneumoniae, 17 cases of negative. Sensitive antibiotics were used for antibacterial treatment based on bacterial culture results. If the result was negative, antibiotics were chosen for treatment empirically. Routine X-ray, CT scan and MRI were taken to evaluate the extent and scope of bone infection.

#### Operation method

After anesthesia, an appropriate incision was selected to expose the lesion. Thorough debridement was performed around the local lesion. All inflammatory tissues including dead bones, scars and pus were removed completely. In this process, the standard we following was to remove the infected bone until healthy bone tissue reached spotting bleeding, which was called the “chili sign” [12]. If the patient had intra-articular infections before surgery, thorough debridement should be performed in the articular cavity first, and closed irrigation or vacuum sealing drainage was used to control the infection. The retention of the internal fixation depends on the degree of infection and whether the existence of the internal fixation can provide effective fixation. Large amounts of hydrogen peroxide and normal saline were used to wash the wound. Then, the wound was soaked with iodophor for 10 min. After replacing surgical drapes and gloves, we mixed calcium phosphate 5 ml powder (Rebone Ltd, China) with vancomycin 0.5 g and gentamicin 2 ml to form antibiotic artificial bone granules. The antibiotic artificial bone granules were placed and dried for 15 min, then filled into the lesion evenly. External fixation was applied timely according to the stability of the fracture end. Drainage tube was placed and wound was sutured [12].

#### Postoperative treatment

Routine anticoagulant and analgesic therapy was performed after surgery. According to the results of preoperative bacterial culture, sensitive antibiotics were injected intravenously for 2 weeks, and then switched to oral antibiotics for 4 weeks. If the results of intraoperative bacterial culture are inconsistent with the preoperative results, antibiotics were adjusted based on intraoperative bacterial culture results. Dressing changes are performed regularly. Wound and drain conditions were monitored. Drainage tube was removed timely.

At present, there is no exact diagnosis and treatment evaluation standard for infection after internal fixation of tibial plateau fractures. We consulted relevant literature and evaluated the efficacy according to whether the fracture healing and infection control. Local wound conditions and laboratory indicators (including C-reactive protein, Erythrocyte sedimentation rate, White blood cells) were recorded to assess infection control. Infection control includes: disappearance of the systemic inflammatory symptoms, wound healing, no local redness, swelling and pain, indexes of CRP, ESR, and WBC are return to normal level. Infection recurrence includes: patients still have fever, local redness, swelling, pain, sinus, poor wound healing, and abnormal CRP, ESR, and WBC indicators. Radiographs were performed to evaluate the healing of the fractures. HSS was used to assess the patients’s joint function.

## Results

A total of 32 patients who met the inclusion criteria were included in this study. There were 18 males and 14 females, aged from 23 to 70 (average 49.66 ± 10.49), 19 cases of the left side and 13 cases of the right side. Seven patients of open fractures and twenty-five patients of closed fractures. All fractures were due to some forms of trauma (falling injury in 18 patients, traffic trauma in 11 patients, crushing injury in 3 patients). The interval between the infection and the first operation were 1 ~ 12 weeks (average 4.69 ± 3.52 weeks). Among them, 7 cases were medial incision infection, 4 cases were lateral incision infection, 21 cases were bilateral incision infection, and 3 cases also had intra-articular infection. Routine examinations were performed after admission, the average preoperative white blood cell (WBC) was (9.07 ± 3.53) × 10^9^/L, the averag preoperative C-reactive protein(CRP) was (35.63 ± 34.65) mg/L, and the preoperative erythrocyte sedimentation rate (ESR) was (47.09 ± 28.03) mm/ h. The basic information of patients was shown in Table 1.

**Table 1:** Patients demographics

Variables	
No.of cases	32
Sex (Male/Female)	18/14
Mean age (years)	49.66 ± 10.49
Location	
Left side	19
Right side	13
Initial trauma	
Traffic trauma	11
Falling injury	18
Crush injury	3
Open fracture	7
Closed fracture	25
The interval between the infection and the first operation(weeks)	4.69 ± 3.52
Infection of the incision	
Medial incision infection	7
Ateral incision infection	4
Bilateral incision infection	21
Inflammatory indicators before surgery	
WBC	(9.07 ± 3.53) × 10^9^
CRP	35.63 ± 34.65
ESR	47.09 ± 28.03

Data shown as number or mean ± standard deviation. *P* < 0.05, significant difference

*WBC* white blood cell

*CRP* C-reactive protein

*ESR* erythrocyte sedimentation rate

**Fig. 1:**
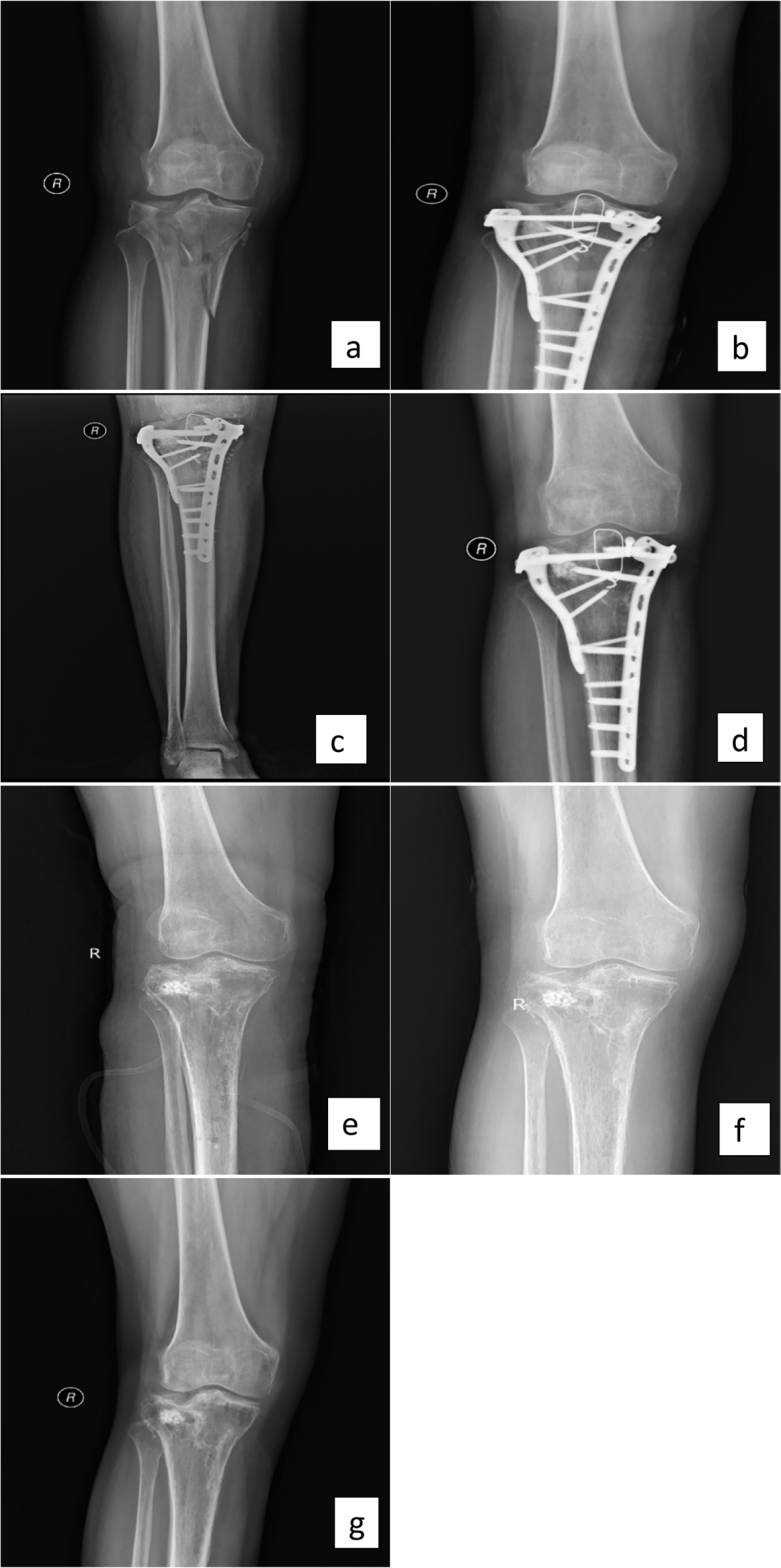
Typical case

**Fig. 2:**
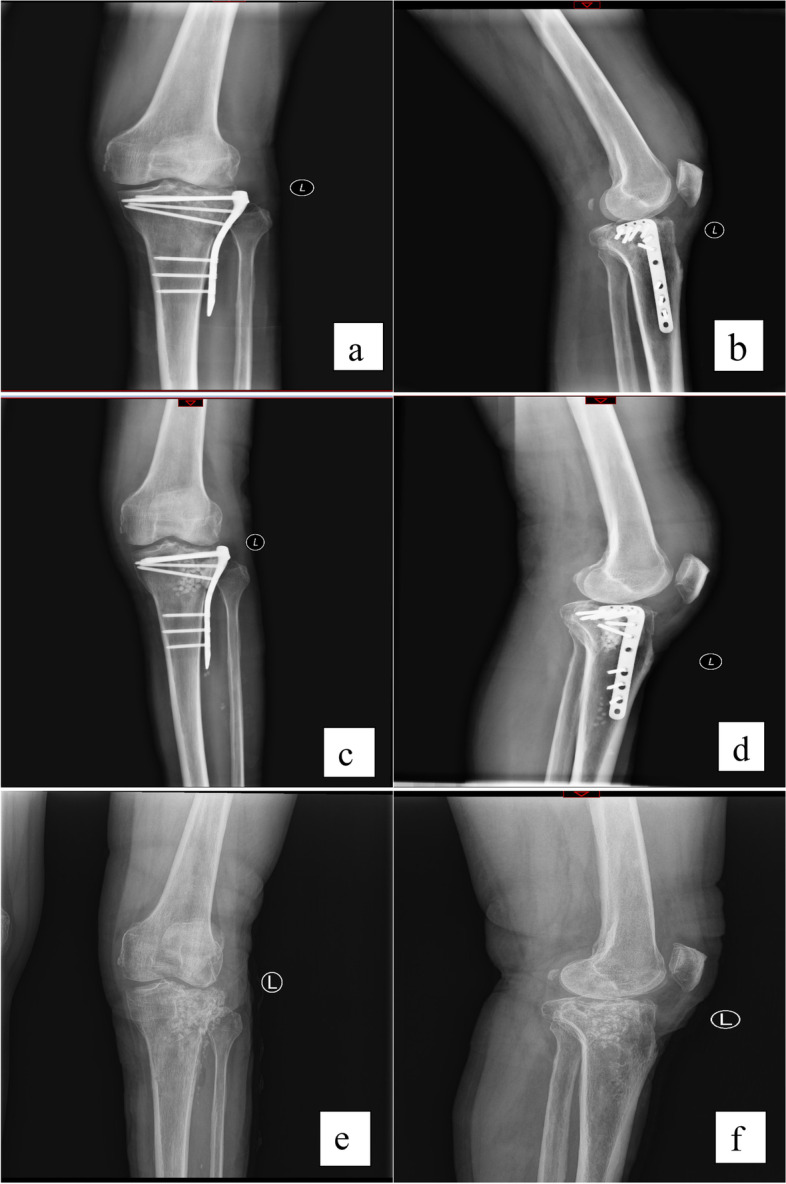
Typical case

All operations were completed successfully without any other complications such as nerve and blood vessel damage. The operation time was 55 ~ 210 min (average 109.22 ± 38.98 min), and intraoperative blood loss were 50 ~ 300 ml (average 157.81 ± 81.43 ml). On the basis of thorough debridement and implanting of antibiotic artificial bone, the internal fixation of 18 patients were tried to be kept and the internal fixation of 14 patients were removed completely. In order to provide effective fixation, 14 patients also received external fixation. Due to intra-articular infection, thorough debridement and irrigation were performed first in 3 patients. Thirty-two patients were followed up for 12 ~ 82 months (average 36.09 ± 19.47 months). The redness, swelling and pain of pin site occurred in 2 patients, which returned to normal after applying antibiotics and continuous dressing change. One patient retained the internal fixation during the first-stage operation. Redness and swelling of incision, subcutaneous undulation occurred after two months. In order to avoid the recurrence of infection, the internal fixation was removed completely and antibiotic artificial bone was filled again. The infection was controlled and fracture healed. Four patientss’ wounds could not be closed directly due to soft tissue defect and was covered with skin flap. After the first-stage operation, 12 patients received second-stage autologous iliac bone grafting due to residual bone defects and poor healing of the fracture end. The bone healing time was 4 ~ 16 months (average 7.31 ± 2.79 months). Inflammatory indicators including CRP, ESR, and WBC returned to normal levels within 2 to 10 weeks (average 4.97 ± 2.58 weeks). The HSS of all patients were 54 ~ 86 points (average 73.06 ± 8.44 points) at the last follow-up. The clinical outcomes of patients were shown in Table 2. Typical cases are shown in the Figs. 1 and 2.

**Table 2:** Summary of clinical outcomes of patients

Variables	
Operation time (min)	109.22 ± 38.98
Intraoperative blood loss (ml)	157.81 ± 81.43
Retention of internal fixation	18
Removal of internal fixation and application of external fixation	14
Follow-up time (month)	36.09 ± 19.47
Bone healing time (month)	7.31 ± 2.79
Hospital for Special Surgery knee score(HSS)	73.06 ± 8.44
Time for inflammatory indicators returned to normal levels (week)	4.97 ± 2.58

Data shown as number or mean ± standard deviation. P < 0.05, significant difference

A 55-year-old female patient with fracture of tibial plateau due to fall damage. Ten days after internal fixation, there was a lot of fluid exudation in the surgical incision and the wound could not heal. The patient was diagnosed with infection after internal fixation of tibial plateau fracture. a: After injury, X-Ray showed tibial plateau fracture. b: After undergoing internal fixation surgery. c: Two months after antibiotic artificial bone implantation, granular high-density shadows can be seen around the internal fixation. d: Eight months after operation, the fracture healed well. We prepared to remove internal fixation. e: Eight months after operation, internal fixation was removed. f: Eleven months after operation, fracture healed well. g: Twenty-five months after operation, fracture healed well.

A 69-year-old male patient with fracture of tibial plateau due to car accident. Twelve days after internal fixation, there was a lot of fluid exudation in the surgical incision and the wound could not be healed. The patient was diagnosed with infection after internal fixation of tibial plateau fracture. a.b. Before receiving antibiotic artificial bone implantation. c.d.Ten days after antibiotic artificial bone implantation, granular high-density shadows can be seen around the internal fixation. e.f. Five months after operation, both the internal fixation and the artificial bone were removed. The patient underwent additional bone grafting surgery.

## Discussion

The incidence of infection after internal fixation of tibial plateau fractures is higher than other parts significantly. Researchs found that open fractures, operation time and smoking are high risk factors for infection [13, 14]. However, there is still no consensus on the treatment of infection after internal fixation of tibial plateau fractures, especially the preservation of internal fixation. Clinicians have different opinions on this issue. Parkkinen et al. believed that the internal fixation should be removed first. Thorough debridement, repeated lavage, systemic or local antibiotics, and flap coverage should be used for treatment according to the patient's condition [15, 16]. Patzakis et al. believed that the stability of the broken end should be considered first, and advocated that the internal fixation should only be removed when the fracture has healed or the internal fixation failed [17]. In our opinion, whether to retain internal fixation should take into account the occurrence time of postoperative infection, the degree and scope of infection, the stability and healing of fracture end, the therapeutic effect of antibiotics, the patient's physical condition and the patient's own will. In the case of retaining internal fixation, if the stability of the fracture end can be maintained, infection was controlled until the fracture healing, which is beneficial to the preservation of knee function. If the infection is severe, the internal fixation failed after debridement, and the broken end is unstable, external fixation should be performed in time. Functional exercise should be carried out as soon as possible after infection control and fracture healing [18]. In our study, the criteria of retaining internal fixation: ⑴Thorough debridement can be performed at the site of infection. ⑵Internal fixation does not fail after debridement. ⑶Bone plate and bone surface fit closely. In such cases, internal fixation can provide more effective fixation of the fracture end. ⑷Local soft tissue condition is good. ⑸The patient is at an early stage of infection. ⑹The fracture has not healed. The criteria of removing internal fixation: ⑴In the case of internal fixation, thorough debridement cannot be performed. ⑵Internal fixation fails after debridement. ⑶The patient is in the stage of delayed infection, and the fracture has healed or partially healed. ⑷The infection is severe and extensive.

The first issue is to control the infection for the treatment of infection after internal fixation of tibial plateau fractures. The research found that early infection after internal fixation of tibial plateau fractures was mostly surgical incision infections [19]. Thorough debridement, adequate drainage and the application of antibiotics can make infection control rate reached 71% while retaining internal fixation [20]. Internal fixation as a metal foreign body, bacteria can form a biofilm on its surface. The resulting barrier effect and low metabolic state of bacteria will make bacteria hard to be killed by antibiotics [21]. However, for the early infection after internal fixation of tibial plateau fracture, the fracture has not yet healed, and the stability provided by internal fixation is also very important for infection control. Therefore, we tried to retain the internal fixation for patients who meet the retention criteria, and then tried to reduce the degree and scope of infection through thorough debridement and application of antibiotics. Even if the infection cannot be eliminated completely, this method also can ensure the infection will not develop further. We tried to make the fracture heals in the case of infection and turn the fracture combined with infection into separate infection. The difficulty of follow-up treatment will be reduced greatly [22]. In this study, the internal fixation of 18 patients were tried to be preserved. Except for 1 patient with recurrence of infection, the other 17 patients controlled the infection successfully until the fracture healed. The internal fixation of 14 patients was removed completely in the first-stage operation due to severe infection, and the postoperative infection was controlled. Late infection will affect the healing of the fracture and cause infectious nonunion. In order to control infection better, the internal fixation is often chosen to be removed. If the fracture is still in the process of healing, internal fixation is effective after thorough debridement and antibiotic therapy is effective, which can still be selected to control the infection until the fracture healing under circumstance of retaining internal fixation. However, it is necessary to pay close attention to the patient's condition during this process [12]. Once the infection is not controlled effectively or even shows signs of recurrence, it is necessary to remove the internal fixation immediately and take appropriate measures to control the infection. In this study, due to the wide range of infection and poor healing of the broken end, the internal fixation of 14 patients with late infection was removed completely on the basis of thorough debridement, antibiotic artificial bone was implanted and external fixation was used for fixation.

The application of antibiotics has always played a very important role in the treatment of bone infection. Shen applied antibiotic bone cement to control the local infection after thorough debridement, and then performed bone grafting to restore the local bone defect after 6 ~ 8 weeks [5]. The infection control rate reached 90%, but the infection recurrence rate after the first stage of this method was higher, multiple debridements are required. Furthermore, antibiotic bone cement cannot be degraded in the body and require second operation to remove [23]. In order to solve this problem, calcium sulfate is used widely in clinical practice as a new type of antibiotic loading material. The substance can be degraded completely in the human body, and loaded antibiotics can be released within 6 ~ 8 weeks to maintain effective local bactericidal concentration of the lesion. Jiang et al. reported that using vancomycin calcium sulfate to treat chronic osteomyelitis of calcaneus [24]. Although the infection was controlled, the incidence of aseptic wound exudation reached 32%. Ruan et al. reported that anti-infection treating after internal fixation of tibial plateau fractures using antibiotic-impregnated calcium sulfate led to aseptic wound exudation incidence reduce to 3% [22]. He believed that persistent wound exudation was related to the massive implantation of calcium sulfate. The researchers found that the release rate of antibiotics loaded with calcium sulfate in the body is relatively stable and wouldn’t represent explosive release similar to bone cement. However, the support strength provided by this substance is insufficient and the osteoinductive ability is weak. The degradation process will produce a lot of water, which may aggravate local infection and limit its clinical application range [25]. Therefore, in this study, the antibiotic sustained release system is a mixture of calcium phosphate with good biocompatibility and antibiotics such as vancomycin. Previous researches indicated calcium phosphate can achieve efficient adsorption of drugs through hydrogen bonding and electrostatic attraction, which can reduce the explosive release effectively at the initial stage after implantation. The local drug concentration can reach more than 4 times of the systemic administration and the effective antibacterial concentration can be maintained locally for 6 ~ 8 weeks [26–28]. Calcium phosphate can also promote activities of osteoblast differentiation and angiogenesis. With the development of bone immunology, calcium phosphate has also been found to have significant immunomodulatory function. Wang et al. discovered that calcium phosphate and its degradation products can promote macrophages to secrete inflammatory cytokines and growth factors, which play a positive role in the migration and osteogenic differentiation of mesenchymal stem cells [29]. Therefore, the use of calcium phosphate loaded with antibiotics to treat infection after internal fixation of tibial plateau fractures can not only plug the dead space and release sensitive antibiotics slowly to control infection, but also induce the repair of bone defects and promote bone healing. In this study, all 32 patients were implanted with antibiotic artificial bone during the first-stage operation, and 31 patients controlled the infection successfully. Due to poor infection control, antibiotic artificial bone was implanted again in one patient, and postoperative infection was controlled effectively. After implanting calcium phosphate loaded with antibiotics, the infection was controlled and the fracture healed well in 20 patients without secondary bone grafting, which accelerated the recovery of patients greatly and helped preserving the function of the affected limb.

In the process of applying antibiotic artificial bone to treat infection after internal fixation of tibial plateau fractures, the use of internal fixation and external fixation should be selected flexibly according to the actual situation of the patient under the guidance of the principles of controlling infection, promoting fracture healing, and maintaining joint function. In the process of treatment, we believe that the following points should be noted: ⑴Debridement is very important. In this process, sequestrum and infected tissues should be removed completely to provide a suitable microenvironment for infection control and bone repair. ⑵The implantation of calcium phosphate should be filled fully without leaving gaps, especially around the internal fixation. ⑶The stability of the fracture end is important for infection control and fracture healing. If the internal fixation failed, external fixation should be employed in time. ⑷Due to the special anatomy of the tibial plateau, soft tissues should be protected as much as possible during the operation. If the wound cannot be closed directly, skin flaps should be used in time. ⑸Functional exercise should be performed as soon as possible after the operation, which can preserve the joint function of the patient to the greatest extent.

This study is limited in its small case number, retrospective study design and lack of control group, which may reduce the persuasiveness of research conclusions. It is necessary to introduce a larger sample of randomized controlled trials to enhance the conviction of the conclusions.

## Conclusion

Implantation of antibiotic artificial bone, retention or removal of internal fixation according to infection and fracture healing, application of external fixation timely is an effective method to treat infection after internal fixation of tibial plateau fractures, which can control infection effectively, promote fracture healing and promote functional recovery.

## Data Availability

The datasets generated and/or analysed during the current study are not publicly available due to limitations of ethical approval involving the patient data and anonymity but are available from the corresponding author on reasonable request**.**
